# Study on the effect of intervention on postpartum psychological improvement and body recovery of caesarean section women based on information‐motivation‐behavioural skills model

**DOI:** 10.1002/nop2.1720

**Published:** 2023-03-12

**Authors:** Linjun Xu, Li Fang, Qianjuan Xu, Danhong Shen

**Affiliations:** ^1^ Department of Surgery Room Hangzhou Women's Hospital Hangzhou China

**Keywords:** caesarean section, emotional state, information‐motivation‐behaviour model, psychological intervention

## Abstract

**Aim:**

To explore the effect of information‐motivation‐behavioural skills (IMB) model on psychological and emotional state of caesarean section (CS) women.

**Design:**

Control‐group patients got routine nursing during the preoperative, intraoperative and postoperative periods. Observation‐group patients received routine nursing combined with the IMB model that included specific informational, motivational and behavioural interventions.

**Methods:**

Pregnant women (*n* = 102) who underwent CS in Hangzhou Women's Hospital were randomly divided into Control and Observation‐groups (*n* = 51 each). Self‐rating anxiety scale (SAS), self‐rating depression scale (SDS) and general self‐efficacy scale (GSES) scores, complication rate, fatigue severity scale (FSS) and Edinburgh postpartum depression scale (EPDS) scores were compared.

**Result:**

Interventions lowered SAS and SDS scores, significantly lower in the Observation‐group (*p* < 0.05), and increased GSES scores, significantly higher in the Observation‐group (*p* < 0.05). Observation‐group had less complications (4.08% vs. 17.39% in the Control‐group; *p* < 0.05), and decreased FSS and EPDS scores (*p* < 0.05).

## 1 INTRODUCTION

Caesarean section (CS) is the most frequently performed obstetric surgery. In recent years, the rate of CS has been increasing (Antoine & Young, 2020). Compared with vaginal delivery, CS is associated with a stronger stress response in pregnant women, which can not only increase the operation risk, but also affect the postpartum recovery (Madsen et al., 2013; Molgora et al., 2020). Studies have suggested that the adverse psychological and emotional state of mothers during pregnancy and childbirth can affect the neurobiology and behaviour of the offspring, and this effect may continue until adulthood (Sandall et al., 2018). Therefore, in recent years, the emphasis has been placed on the psychological intervention during the perioperative period in women who are about to undergo CS (Che et al., 2020). On the basis of a comprehensive analysis of patients' psychological and emotional states, targeted psychological intervention measures can effectively improve the adverse psychological mood and further improve the safety of delivery (Hemanth Kumar et al., 2014). The IMB model was first proposed by Fisher and other scholars in 1992 in the process of studying high‐risk behaviours associated with AIDS. The model suggests that the occurrence of the disease‐prevention behaviour is affected by whether the patient has obtained sufficient information, motivation, and skills. Based on the IMB model, intervention can promote self‐motivation and healthy behaviour, and encourage active cooperation with clinical intervention (Ali et al., 2020; Chu et al., 2019). The IMB model not only provides a framework for analysing the influencing factors of the behaviour, but also outlines the impact of various factors on the behaviour of patients, helping to predict it (Ntoumanis et al., 2021). At present, the IMB mode of intervention is mainly applied to patients with AIDS and diabetes, while the reports of applying it to mothers undergoing CS are limited (Bakır et al., 2021; Dubov et al., 2018). Therefore, this study evaluated the efficiency of the IMB model intervention in the perioperative period of CS and discussed its effect on the improvement of postpartum psychological and emotional state and postpartum recovery.

## 2 MATERIALS AND METHODS

One hundred and two pregnant women who underwent caesarean section in Hangzhou Women's Hospital from January 2020 to June 2021 were randomly divided into Control‐group and Observation‐group, with 51 cases in each group. Two patients in the Observation‐group withdrew halfway. All pregnant women met the indications of caesarean section or the requirements of pregnant women for caesarean section.

The medical ethics Association of Hangzhou Women's Hospital has approved the study (Approval number: 2021‐K‐10‐09, date: 2021‐10‐19). Inclusion criteria were as follows: (1) Voluntary choice of caesarean section to terminate the pregnancy and successful completion of a caesarean section without death. (2) The interval between the caesarean section and the last caesarean section of more than 18 months. (3) Met the criteria for a caesarean section. (4) Aware of the study and cooperates with informed consent. Exclusion criteria were as follows: (1) History of mental illness. (2) Have cognitive and language communication barriers. (3) Suffering from other serious basic diseases, organ dysfunction and malignant tumours. (4) Withdrawal from the study.

### 2.1 Treatment protocol

#### 2.1.1 Control‐group

All the patients in the Control‐group received routine nursing, and the routine nursing was as follows.

- Preoperative and intraoperative care: The nurses introduced the basic procedure of CS, the environment of the operating room and the ward to the puerperae, and informed them of precautions and cooperation points, calmed their anxiety, encouraged them, patiently listened and responded to maternal complaints. Close interaction was maintained during the operation, through limb and eye contact, which helped to build a trusting relationship between the surgeons, nurses and the puerperae.
- Postoperative care: Patients were immediately informed of the successful operation after the surgery. Mother‐infant contact was established as soon as possible. The blood pressure, heart rate, pulse, vital signs, uterine contraction and vaginal bleeding of the puerperae should be closely observed, and the doctors should be notified in time if there is any abnormality. The puerperae should adopt a semi‐recumbent position 24 h after CS, which can promote the discharge of lochia. The urinary catheter was indwelled for 24 h after CS, and the puerperae were instructed to urinate as soon as possible after extubation. The puerperae were encouraged to turn over frequently and get out of bed as soon as possible.

#### 2.1.2 Observation‐group

Patients in the Observation‐group received the same routine nursing as the Control‐group (see above). In addition, a special IMB mode intervention team was set up and included one doctor and three nurses. The team carried out IMB mode intervention on the Observation‐group for 2 months. All team members have been trained by IMB professionals. The key points of the IMB mode intervention include the following:

- Information intervention: After the patients were admitted to the hospital, the pain questionnaire and face‐to‐face communication were used to understand the cognition level of the patients, and health education was carried out based on these data. In addition to introducing the basic knowledge of routine nursing to patients (operation‐related knowledge, the environment of the operating room and the ward, precautions and cooperation points), nurses also informed the patients about the prevalence of CS, CS procedure, CS‐related complications, as well as the key points of rehabilitation after CS, which allowed patients to have a more comprehensive understanding of CS. Relevant knowledge was compiled into a health education brochure, which was distributed to the parturients for independent reading. The key points of the health education brochure were listed in Table 1.
- Motivational intervention: (A) Unconscious intervention. The main goal of unconscious intervention is to establish a trust relationship with pregnant women, communicate with them cordially, encourage them to share their inner thoughts, listen patiently, express understanding and answer their questions in detail. In this case, it helped to build a trusting relationship between the nurses and the pregnant women. (B) Intention period: pregnant women were encouraged and guided to learn about the CS, with a correct understanding of the principles of CS cooperation and postpartum recovery, to improve their health awareness. (C) Preparation period. Pregnant women were helped to set up perioperative cooperation objectives and programs and were given personalized suggestions and guidance. (D) Change period. The relevant knowledge of CS was reviewed, and maternal understanding of knowledge implementation was assessed. (E) Maintenance period. Pregnant women were encouraged to make full use of the existing resources and to maintain a long‐term healthy behaviour.
- Behavioural intervention: (A) Before the operation, patients were informed in detail of the discomfort and complications that may occur during the operation, and operation safety and prognosis were discussed to make them cooperate actively. (B) After CS, in addition to the postoperative care mentioned in the routine nursing, the women were given behavioural skills training on how to rapidly recover from CS, enlisted social support from family members and received self‐regulation strategies. (1) Rapid recovery behavioural skills: 6 h after CS, the puerpera performed straight leg lifting exercises to enhance abdominal muscle tension, six times per minute. Three days after delivery, they performed sit‐ups, four times per minute; waist rotation exercises were performed to exercise the muscles of the lower back, six times per minute; squat upright exercise to strengthen leg muscles were done 10 times per minute. Women were guided to walk slowly to exercise the muscle strength of lower limbs, 4–5 min each time. Moreover, the puerperae were taught to exercise the pelvic floor muscles and fascia through the anal contraction exercise: the puerperae took a free position, lifted the anus, urethra and perineum as much as possible, contracted once every 5–10 s, rested for 5–10 s and then repeated the training for 5 min, three times a day, until the end of puerperium. (2) Social support from family members: ① Family members were guided to massage the lower abdomen of the puerpera after CS, with even force in a clockwise direction, from light to heavy massage, which can help to promote uterine contraction. ② Six hours after CS, the responsible nurse evaluated the situation of the women, provided semi‐liquid food rich in protein, vitamins, minerals and cellulose according to the personal situation of the women, and formulated a special and reasonable healthy diet plan for women with special needs, to adequately supplement the nutrition and energy. Family members were encouraged to strictly follow the nurse's requirements to give a nutritional supplement to the puerpera. ③ Family members received guidance on breastfeeding and helped mothers breastfeed for the first time. (3) Self‐regulation strategies: ① Self‐body management: Puerpera should pay close attention to their physical condition and timely report any discomfort after CS, and perform the rapid recovery behavioural tasks accordingly. ② Caesarean incision care: since it takes about 5 weeks for the incision to heal after CS, the mothers were provided with guidelines for incision health management and were encouraged to pay attention to exudate, infection and cleanliness of the incision. ③ Timely contraceptive measures: women were provided with the information that sexual intercourse should not be started until 42 days after the delivery and after the lochia has completely cleared.

**TABLE 1:** Key points of the health education brochure.

Sections	Key points
Section 1. What is caesarean section?	Definition of CS. Differences between CS and normal vaginal delivery. When to choose CS? The prevalence of CS.
Section 2. Is a caesarean section safe?	Advantages and disadvantages of CS. CS‐related complications.
Section 3. What is the procedure of caesarean section?	Patient consent. Pre‐CS preparation: ⚬ Systemic examination and necessary laboratory tests; ⚬ Disinfection measures; ⚬ Placement of urinary catheter; Intravenous drip; Preoperative anaesthesia. Caesarean procedure: ⚬ Open the abdominal wall; ⚬ Hysterotomy; ⚬ Pull out the fetus; ⚬ Stripping afterbirth; ⚬ Suture the uterus; ⚬ Suture the abdominal wall.
Section 4. Precautions before, during and after caesarean section.	Preoperative precautions. Intraoperative precautions. Postoperative precautions.
Section 5. Recovery guidance after caesarean section.	Pain management. Caesarean incision care. Emotion management. Dietary considerations. Precautions for exercise.

Medical records of all the patients included assessment of the following observation indexes: SAS and SDS were used to evaluate the psychological and emotional state of pregnant women before and after the intervention. There were 20 items in SAS, the frequency of symptoms was mainly assessed by the 4‐level scoring method, the higher the total score, the higher the degree of anxiety. The scale has been revised in China, according to the results of the Chinese norm, and the SAS standard was divided into 50 points, of which 50–59 points are mild anxiety, 60–69 points are moderate anxiety, and 70 points and above are severe anxiety (Grigoriadis et al., 2018). There are 20 items in the SDS, the rough score is obtained by adding the scores of the 20 items using the 4‐level scoring method. The rough score is multiplied by 1.25 and the integer part is taken to get the standard score; the higher the standard score, the more serious the degree of depression. According to the results of the Chinese norm, the SDS standard was divided into 53 points, 53–62 points for mild depression, 63–72 points for moderate depression, and ≥73 points for severe depression (Zung, 1965). The general self‐efficacy scale (GSE) (Schwarzer & Jerusalem, 1995) was used to evaluate the self‐efficacy of pregnant women before and after the intervention. The scale has 10 items. For each item, the following answers are used: Not at all true (one point), hardly true (two points), moderately true (three points), exactly true (four points), with a total score of 10–40 points. Higher scores indicate better self‐efficacy (Luszczynska et al., 2005). The incidences of complications such as puerperal infection, postpartum haemorrhage and amniotic fluid embolism in the two groups were analysed. Before intervention and 2 weeks postpartum, the postpartum recovery of the two groups was evaluated by FSS and EPDS. There were nine items in FSS, and each item ranged from ‘completely disagree’ to ‘fully agree’ (score 0–7 respectively), with a total score of 63. Higher score indicated more serious fatigue (Learmonth et al., 2013). EPDS contains a total of 10 problems, each with four options scored at level 0–3, with a total score between 0 and 30. A score ≥10 indicates depression, and higher scores correlate with the severity of depression (Badr et al., 2018).

### 2.2 Statistical analysis

The data of this study were processed by SPSS22.0. [*n* (%)] representing the counting data, the test methods used were *χ* ^2^ or Fisher's exact test. The measurement data were tested for normality by Shapiro–Wilk test, and ($\bar{\chi }\pm s$) was used to represent the measurement data that conformed to normal distribution and homogeneity of variance test. Paired *t*‐test was used for intra‐group comparison, and án independent sample *t*‐test was used for inter‐group comparison for measurement data. The significance level was set at 0.05.

## 3 RESULTS

In total, 102 women were included in the study and randomly divided into two equal groups (*n* + 51 in each group). The average age of the Control‐group was 28.17 ± 3.79 years old. The average gestational age was 39.86 ± 0.96 weeks. The group included 28 primiparas and 23 multiparas. There were 12 cases of premature birth, 37 cases of full‐term birth and two cases of overdue birth. The causes of the caesarean section were as follows: 13 cases of pregnancy complications, 19 cases of fetal distress, 17 cases of voluntary request, and two cases for other reasons. The average age of the Observation‐group was 28.61 ± 3.77 years old. The average gestational age was 39.92 ± 0.97 weeks, with 34 primiparas and 15 multiparas. There were eight cases of premature birth, 38 cases of full‐term birth and three cases of overdue birth. Causes of the caesarean section included 13 cases of pregnancy complications, 14 cases of fetal distress, 19 cases of voluntary request and three cases—other reasons. There was no significant difference between the two groups in terms of age, gestational age, delivery and other general data (*p* > 0.05), which was comparable (Table 2). Before the intervention, there was no significant difference in SAS, SDS and GSEs scores between the two groups (*p* > 0.05), after the intervention, while the SAS and SDS scores of both groups decreased, the Observation‐group had significantly lower SAS and SDS scores as compared to the Control‐group (*p* < 0.05), the GSEs scores of the two groups increased after intervention, the GSEs scores of the Observation‐group was significantly higher than the Control‐group (*p* < 0.05) (Table 3); The incidence of complications in the Observation‐group was 3.70%, lower than 14.81% in the Control‐group (*p* < 0.05) (Table 4). There were no women with more than one complication in our study; Before the intervention, there was no significant difference in the scores of FSS and EPDS between the two groups (*p* > 0.05), but on the 14th day after delivery, the scores of FSS and EPDS decreased in both groups, and patients in the Observation‐group showed significantly lower FSS and EPDS scores than the Control‐group (*p* < 0.05) (Table 5).

**TABLE 2:** Comparison of basic data of two groups of parturients [*n* (%), $\bar{\chi }\pm s$].

	Age (years)	Gestational weeks	Fetus number		Condition of delivery			Causes of caesarean section			
			Primipara women	Parturient women	Premature	Full‐term	Overdue production	Pregnancy complications	Fetal distress	Voluntary request	Others
Control‐group (*n* = 51)	28.17 ± 3.79	39.86 ± 0.96	28 (54.9)	23 (45.1)	12 (23.5)	37 (72.5)	2 (4.0)	13 (25.5)	19 (37.3)	17 (33.3)	2 (3.9)
Observation‐group (*n* = 49)	28.61 ± 3.77	39.92 ± 0.97	34 (69.4)	15 (30.6)	8 (16.3)	38 (77.6)	3 (6.1)	13 (26.5)	14 (28.6)	19 (38.8)	3 (6.1)
*t*/Fisher	−0.577	−0.287	2.226		0.974			1.029			
*p*‐value	0.566	0.774	0.136		0.615			0.794			

**TABLE 3:** Comparison of SAS, SDS and GSEs scores between the two groups before and after the intervention ($\bar{\chi }\pm s$, score).

Group (*n*)	SAS		SDS		GSES	
	Before intervention	After intervention	Before intervention	After intervention	Before intervention	After intervention
Control‐group (*n* = 51)	62.31 ± 6.98	52.39 ± 6.94*	59.49 ± 6.75	46.37 ± 6.25*	22.56 ± 2.78	27.27 ± 2.69*
Observation‐group (*n* = 49)	61.98 ± 6.46	42.83 ± 6.72*	60.67 ± 6.61	42.20 ± 5.97*	22.18 ± 2.52	30.63 ± 2.79*
*t*	0.247	6.990	−0.885	3.406	0.724	−6.111
*p*‐value	0.805	<0.001	0.378	0.001	0.471	<0.001

Abbreviations: SAS, self‐rating anxiety scale; SDS, self‐rating depression scale.

* Indicates the comparison with that before the intervention, *p* < 0.05.

**TABLE 4:** Comparison of postpartum complications between the two groups [*n* (%)].

Group	*n*	Complication			Total
		Puerperal infection	Postpartum haemorrhage	Amniotic fluid embolism	
Control‐group	51	5 (9.8)	3 (5.9)	1 (1.9)	9 (17.6)
Observation‐group	49	1 (2.0)	1 (2.0)	0 (0.00)	2 (4.0)
*χ* ^2^	‐	‐	‐	‐	4.697
*p*‐value	‐	‐	‐	‐	0.030

**TABLE 5:** Comparison of postpartum recovery between the two groups ($\bar{\chi }\pm s$, score).

Group (*n*)	FSS		EPDS	
	Before intervention	After intervention	Before intervention	After intervention
Control‐group (*n* = 51)	50.21 ± 5.02	35.25 ± 4.44*	18.62 ± 3.44	10.98 ± 2.98*
Observation‐group (*n* = 49)	49.45 ± 5.10	25.30 ± 4.42*	18.96 ± 2.95	6.73 ± 2.05*
*t*	0.757	11.207	−0.516	8.263
*p*‐value	0.451	<0.001	0.607	<0.001

Abbreviations: EPDS, Edinburgh postpartum depression scale; FSS, fatigue severity scale.

* Indicates the comparison with that before the intervention, *p* < 0.05.

## 4 DISCUSSION

It has been reported that the prevalence of maternal postnatal anxious symptomatology was 34.5% at 1–24 weeks postpartum, and 30.8% at >24 weeks postpartum, which seriously affects maternal mental health and postpartum quality of life (Cena et al., 2021). Our results showed that the SAS and SDS scores of patients that received the IMB model of nursing were significantly lower than those who received routine nursing. We considered the reasons as follows: (1) Patients received IMB model gained more detailed knowledge of preparations of CS and postpartum rehabilitation than patients received routine nursing, which may be helpful to reduce the psychological pressure of the puerperae on CS and enhance the confidence of the puerperae in rehabilitation after CS. (2) The motivational intervention stage helped the puerperae to establish a correct understanding of CS, which was conducive to strengthening the awareness of maternal pursuit of health, correcting the patient's mentality from the psychological level, and attaching importance to postpartum rehabilitation.

In addition, our study also found that the GSEs score of the Observation‐group was higher than that of the Control‐group. Previous study by Wang et al. (2019) evaluated 165 cases of breast cancer patients in the perioperative period of information and showed that motivation and behavioural skills model significantly improved patient's compliance, promoted their health behaviour, and thereby improved their self‐efficacy. In our study, the IMB model includes comprehensive health education for patients in the information stage and introduces the relevant knowledge of caesarean section in detail, improving maternal knowledge level. The motivation stage focused on scientific guidance. We established a mutual trust relationship with the puerperae at the initial stage, then guided puerperae to set up postoperative rehabilitation goals and implementation plans. Family supports were encouraged to establish a sustainable peripheral environment for postpartum rehabilitation. All these can effectively correct maternal psychological behaviour, and promote better health consciousness and motivation. In the behavioural intervention stage, the perioperative physiological discomfort can be reduced by implementing preoperative and postoperative behavioural interventions (Shirzad et al., 2020). Through the implementation of the above three aspects of intervention measures, pregnant women can have a proper understanding of CS. This, in turn, can alleviate their anxiety caused by insufficient cognition, thereby positively adjusting their emotions, and improving compliance, overall psycho‐emotional state and self‐efficacy (Andrighetti et al., 2017).

Postpartum complications of CS mainly include puerperal infection, postpartum haemorrhage, amniotic fluid embolism, etc (Gonzalo‐Carballes et al., 2020). Studies show that poor psychological and emotional state of pregnant women is a risk factor of postpartum morbidities, since it leads to reduced level of perioperative clinical interventions and subsequent increase in surgery‐related risks and complications (Burke & Allen, 2020; Woodd et al., 2019). Postpartum complications following CS not only affect the physical and mental health of women, but also directly reduce the efficiency of postpartum recovery (Hung et al., 2016). In this study, the incidence of complications in the Observation‐group was lower than that in the Control‐group (*p* < 0.05), suggesting that the self‐care behaviours applied by the IMB model in the perioperative intervention of CS women can reduce the incidence of complications.

Moreover, our study also found that the postpartum FSS and EPDS of the Observation‐group were lower than those of the Control‐group (*p* < 0.05), suggesting that the implementation of IMB model intervention for women, undergoing cesarian section, can further facilitate the efficiency of postpartum recovery. Implementation of IMB model intervention, therefore, can improve psychological and emotional state and self‐efficacy, and allows to actively coordinate necessary perioperative interventions in women undergoing cesarian section. This not only reduces surgery‐related complications but also facilitates and improves the effect of successful postpartum rehabilitation.

This study has several limitations, such as a small sample size, few observation indicators and no long‐term postpartum follow‐up, which may make the conclusion one‐sided and limited. Additionally, the study was performed during the period of the COVID‐19 pandemic which had an impact on the perinatal care system and on the mental health of pregnant women, which may affect the results. Therefore, the follow‐up multi‐center and large‐scale studies are needed to comprehensively analyse the perioperative psychological and emotional state and psychological intervention effect on women undergoing CS.

## 5 CONCLUSION

Compared with routine nursing, nursing intervention based on the IMB model can more effectively improve the postpartum psychological and emotional state of women that are undergoing CS, enhance self‐efficacy, reduce the incidence of complications, and help to promote postpartum recovery.

## AUTHOR CONTRIBUTIONS

LX conceptualized and designed the study; LX and LF did literature search and data collection; QX and DS analysed the data; LX and LF wrote the paper; QX and DS reviewed and edited the manuscript. All authors read and approved the final manuscript.

## FUNDING INFORMATION

The current study was funded by Zhejiang Medicine and Health Science and Technology Plan Project (Grant number: 2019322760).

## CONFLICT OF INTEREST STATEMENT

No conflict of interest has been declared by the authors.

## RESEARCH ETHICS COMMITTEE APPROVAL

The medical ethics Association of Hangzhou Women's Hospital has approved the study (Approval number: 2021‐K‐10‐09, date: 2021‐10‐19).

## Data Availability

The data that support the findings of this study are available from the corresponding author upon reasonable request.

## References

[nop21720-bib-0001] Ali, M. , Sultan, S. F. , Kumar, A. , & Ghouri, N. (2020). Knowledge, attitude and practices of labor analgesia amongst healthcare workers and patients: A single center cross sectional study. Pakistan Journal of Medical Sciences, 36(1), S4–S8. 10.12669/pjms.36.ICON-Suppl.1715 31933599 PMC6943109

[nop21720-bib-0002] Andrighetti, H. J. , Semaka, A. , & Austin, J. C. (2017). Women's experiences of participating in a prospective, longitudinal postpartum depression study: Insights for perinatal mental health researchers. Archives of Women's Mental Health, 20(4), 547–559. 10.1007/s00737-017-0744-7 PMC5511519 28600644

[nop21720-bib-0003] Antoine, C. , & Young, B. K. (2020). Cesarean section one hundred years 1920‐2020: The good, the bad and the ugly. Journal of Perinatal Medicine, 49(1), 5–16. 10.1515/jpm-2020-0305 32887190

[nop21720-bib-0004] Badr, L. K. , Ayvazian, N. , Lameh, S. , & Charafeddine, L. (2018). Is the effect of postpartum depression on mother‐infant bonding universal? Infant Behavior & Development, 51, 15–23. 10.1016/j.infbeh.2018.02.003 29533871

[nop21720-bib-0005] Bakır, M. S. , Birge, Ö. , Karadag, C. , Doğan, S. , & Simsek, T. (2021). Laparoscopic treatment of recurrent and chemoresistant cesarean scar choriocarcinoma. Clinical Case Reports, 9(3), 1457–1461. 10.1002/ccr3.3801 33768867 PMC7981746

[nop21720-bib-0006] Burke, C. , & Allen, R. (2020). Complications of cesarean birth: Clinical recommendations for prevention and management. The American Journal of Maternal Child Nursing, 45(2), 92–99. 10.1097/NMC.0000000000000598 31804227

[nop21720-bib-0007] Cena, L. , Gigantesco, A. , Mirabella, F. , Palumbo, G. , Trainini, A. , & Stefana, A. (2021). Prevalence of maternal postnatal anxiety and its association with demographic and socioeconomic factors: A multicentre study in Italy. Frontiers in Psychiatry, 12, 737666. 10.3389/fpsyt.2021.737666 34658970 PMC8514655

[nop21720-bib-0008] Che, Y.‐J. , Gao, Y.‐L. , Jing, J. , Kuang, Y. , & Zhang, M. (2020). Effects of an informational video about anesthesia on pre‐ and post‐elective cesarean section anxiety and recovery: A randomized controlled trial. Medical Science Monitor, 26, e920428. 10.12659/MSM.920428 32265432 PMC7165245

[nop21720-bib-0009] Chu, J. Y. , Jiang, B. , Gao, Y. P. , Li, L. , Yang, M. J. , Ma, F. F. , & Liu, X. L. (2019). Evaluation on the effect of exclusive breastfeeding among women with primipara, using the information‐motivation‐behavioral skills model intervention model. Zhonghua Liu Xing Bing Xue Za Zhi, 40(12), 1639–1644. 10.3760/cma.j.issn.0254-6450.2019.12.025 32062930

[nop21720-bib-0010] Dubov, A. , Galbo, P. , Altice, F. L. , & Fraenkel, L. (2018). Stigma and shame experiences by MSM who take PrEP for HIV prevention: A qualitative study. American Journal of Men's Health, 12(6), 1843–1854. 10.1177/1557988318797437 PMC6199453 30160195

[nop21720-bib-0011] Gonzalo‐Carballes, M. , Ríos‐Vives, M. Á. , Fierro, E. C. , Azogue, X. G. , Herrero, S. G. , Rodríguez, A. E. , Rus, M. N. , Planes‐Conangla, M. , Escudero‐Fernandez, J. M. , & Coscojuela, P. (2020). A pictorial review of postpartum complications. Radiographics, 40(7), 2117–2141. 10.1148/rg.2020200031 33095681

[nop21720-bib-0012] Grigoriadis, S. , Graves, L. , Peer, M. , Mamisashvili, L. , Tomlinson, G. , Vigod, S. N. , Dennis, C.‐L. , Steiner, M. , Brown, C. , Cheung, A. , Dawson, H. , Rector, N. A. , Guenette, M. , & Richter, M. (2018). Maternal anxiety during pregnancy and the association with adverse perinatal outcomes: Systematic review and meta‐analysis. The Journal of Clinical Psychiatry, 79(5), 17r12011. 10.4088/JCP.17r12011 30192449

[nop21720-bib-0013] Hemanth Kumar, V. , Jahagirdar, S. M. , Athiraman, U. K. , Sripriya, R. , Parthasarathy, S. , & Ravishankar, M. (2014). Study of patient satisfaction and self‐expressed problems after emergency caesarean delivery under subarachnoid block. Indian Journal of Anaesthesia, 58(2), 149–153. 10.4103/0019-5049.130815 24963178 PMC4050930

[nop21720-bib-0014] Hung, H.‐W. , Yang, P.‐Y. , Yan, Y.‐H. , Jou, H.‐J. , Lu, M.‐C. , & Wu, S.‐C. (2016). Increased postpartum maternal complications after cesarean section compared with vaginal delivery in 225 304 Taiwanese women. The Journal of Maternal‐Fetal & Neonatal Medicine, 29(10), 1665–1672. 10.3109/14767058.2015.1059806 26135778

[nop21720-bib-0015] Learmonth, Y. C. , Dlugonski, D. , Pilutti, L. A. , Sandroff, B. M. , Klaren, R. , & Motl, R. W. (2013). Psychometric properties of the fatigue severity scale and the modified fatigue impact scale. Journal of the Neurological Sciences, 331(1–2), 102–107. 10.1016/j.jns.2013.05.023 23791482

[nop21720-bib-0016] Luszczynska, A. , Scholz, U. , & Schwarzer, R. (2005). The general self‐efficacy scale: Multicultural validation studies. The Journal of Psychology, 139(5), 439–457. 10.3200/JRLP.139.5.439-457 16285214

[nop21720-bib-0017] Madsen, K. , Grønbeck, L. , Rifbjerg Larsen, C. , Østergaard, J. , Bergholt, T. , Langhoff‐Roos, J. , & Sørensen, J. L. (2013). Educational strategies in performing cesarean section. Acta Obstetricia et Gynecologica Scandinavica, 92(3), 256–263. 10.1111/aogs.12055 23173712

[nop21720-bib-0018] Molgora, S. , Fenaroli, V. , Cracolici, E. , & Saita, E. (2020). Antenatal fear of childbirth and emergency cesarean section delivery: A systematic narrative review. Journal of Reproductive and Infant Psychology, 38(4), 436–454. 10.1080/02646838.2019.1636216 31271306

[nop21720-bib-0019] Ntoumanis, N. , Ng, J. Y. Y. , Prestwich, A. , Quested, E. , Hancox, J. E. , Thøgersen‐Ntoumani, C. , Deci, E. L. , Ryan, R. M. , Lonsdale, C. , & Williams, G. C. (2021). A meta‐analysis of self‐determination theory‐informed intervention studies in the health domain: Effects on motivation, health behavior, physical, and psychological health. Health Psychology Review, 15(2), 214–244. 10.1080/17437199.2020.1718529 31983293

[nop21720-bib-0020] Sandall, J. , Tribe, R. M. , Avery, L. , Mola, G. , Visser, G. H. , Homer, C. S. , Gibbons, D. , Kelly, N. M. , Kennedy, H. P. , Kidanto, H. , Taylor, P. , & Temmerman, M. (2018). Short‐term and long‐term effects of caesarean section on the health of women and children. The Lancet, 392(10155), 1349–1357. 10.1016/S0140-6736(18)31930-5 30322585

[nop21720-bib-0021] Schwarzer, R. , & Jerusalem, M. (1995). General self‐efficacy scale. In J. Weinman , S. Wright , & M. Johnton (Eds.), Measures in health psychology: A user's portfolio. Causal and control beliefs (pp. 35–37). NFER‐NELSON. 10.1037/t00393-000

[nop21720-bib-0022] Shirzad, M. , Shakibazadeh, E. , Rahimi Foroushani, A. , Abedini, M. , Poursharifi, H. , & Babaei, S. (2020). Effect of “motivational interviewing” and “information, motivation, and behavioral skills” counseling interventions on choosing the mode of delivery in pregnant women: A study protocol for a randomized controlled trial. Trials, 21(1), 970. 10.1186/s13063-020-04865-3 33239038 PMC7687772

[nop21720-bib-0023] Wang, X. , Jia, M. , Li, Y. , Bao, Y. , Zhang, C. , Zhou, C. , Wang, L. , Cao, X. , Jiang, R. , & Li, F. (2019). Validation of an information‐motivation‐behavioral skills model of upper limb functional exercise adherence among Chinese postoperative patients with breast cancer. Breast Cancer, 26(2), 198–205. 10.1007/s12282-018-0911-3 30259414

[nop21720-bib-0024] Woodd, S. L. , Montoya, A. , Barreix, M. , Pi, L. , Calvert, C. , Rehman, A. M. , Chou, D. , & Campbell, O. M. R. (2019). Incidence of maternal peripartum infection: A systematic review and meta‐analysis. PLoS Medicine, 16(12), e1002984. 10.1371/journal.pmed.1002984 31821329 PMC6903710

[nop21720-bib-0025] Zung, W. W. (1965). A self‐rating depression scale. Archives of General Psychiatry, 12, 63–70. 10.1001/archpsyc.1965.01720310065008 14221692

